# Smiley faces and the need for careful planning in trials

**DOI:** 10.1186/1745-6215-16-S2-P208

**Published:** 2015-11-16

**Authors:** Mike Clarke, Lisa Maguire, Helen McAneney

**Affiliations:** 1 Northern Ireland Network for Trials Methodology Research, Queen's University Belfast, Belfast, UK; 2 North West Hub for Trials Methodology Research, University of Liverpool, Liverpool, UK

## Background

Trialists need to plan the data they will collect, how they will do so and how they will analyse it. If data are not collected at the appropriate time, it may be impossible to obtain them later. Inconsistencies in the understanding of participants about the data they are asked to provide may make it impossible to combine it. A lack of advance planning of the analyses may mean that knowledge of the data introduces bias into the reported results.

## Aim and objective

To show the importance of careful planning, by asking people to “draw a smiley face”.

## Methods

Participants in various courses and meetings have been asked to draw a smiley face, and add their name, age and gender, should they wish. Subsequent discussions with the participants highlight how people interpret the instruction in different ways, how the faces vary and how a lack of hypotheses provide opportunities for biased analyses and interpretation. Drawings were categorised and facial features were counted, allowing comparisons between groups of participants.

## Results

By May 2015, nearly 400 drawings had been collected. Preliminary findings show the range of faces that have been drawn, including a large proportion that would be considered as close to the classic smiley face, but also many variants. The presentation will include analyses of the data and the opportunity to consider its relevance to the planning of trials.

## Conclusion

The smiley face experiment illustrates how collecting an outcome measure which might seem relatively simple can become complex without careful planning.

